# Role of Iron in Aging Related Diseases

**DOI:** 10.3390/antiox11050865

**Published:** 2022-04-28

**Authors:** William J. Chen, George P. Kung, Jaya P. Gnana-Prakasam

**Affiliations:** 1 Department of Ophthalmology, Saint Louis University, St. Louis, MO 63104, USA; william.j.chen@health.slu.edu (W.J.C.); george.kung@health.slu.edu (G.P.K.); 2 Department of Biochemistry and Molecular Biology, Saint Louis University, St. Louis, MO 63104, USA

**Keywords:** iron, aging, oxidative stress, chronic diseases, iron chelator

## Abstract

Iron progressively accumulates with age and can be further exacerbated by dietary iron intake, genetic factors, and repeated blood transfusions. While iron plays a vital role in various physiological processes within the human body, its accumulation contributes to cellular aging in several species. In its free form, iron can initiate the formation of free radicals at a cellular level and contribute to systemic disorders. This is most evident in high iron conditions such as hereditary hemochromatosis, when accumulation of iron contributes to the development of arthritis, cirrhosis, or cardiomyopathy. A growing body of research has further identified iron’s contributory effects in neurodegenerative diseases, ocular disorders, cancer, diabetes, endocrine dysfunction, and cardiovascular diseases. Reducing iron levels by repeated phlebotomy, iron chelation, and dietary restriction are the common therapeutic considerations to prevent iron toxicity. Chelators such as deferoxamine, deferiprone, and deferasirox have become the standard of care in managing iron overload conditions with other potential applications in cancer and cardiotoxicity. In certain animal models, drugs with iron chelating ability have been found to promote health and even extend lifespan. As we further explore the role of iron in the aging process, iron chelators will likely play an increasingly important role in our health.

## 1. Introduction

Iron is vital for the transport of oxygen in the hemoglobin of red blood cells and in the myoglobin of muscles. It is also a quintessential component of cytochromes and other proteins and cofactors involved in critical systemic biochemical reactions. Diet is the main source of iron for the body and with a lack of specialized mechanism for excretion of excess iron, most organisms including *Caenorhabditis elegans* [1], *Drosophila melanogaster* [2], *Saccharomyces cerevisiae* [3], *Rattus norvegicus* [4], *Mus musculus* [5], and *Homo sapiens* [6] have been found to accumulate iron as they age. Several studies indicate that iron accumulation further contributes to cellular aging in a multitude of these species [7,8,9,10,11]. Iron supplementation is found to accelerate the aging process by increasing oxidative stress whereas iron restriction slows the aging [12,13]. A recent report on multivariate genome scanning suggests that high levels of iron in the blood are associated with reduced healthy lifespan [14]. Lower iron levels in women before menopause may also be a contributing factor to the increased longevity in women compared to men. Collectively, the evidence indicates a strong positive correlation between iron accumulation and aging.

## 2. Iron Overload and Oxidative Stress

Iron has an inherent ability to exchange electrons with other molecules serving as electron donor and acceptor in the form of ferrous (Fe^2+^) and ferric (Fe^3+^) iron respectively. Hence iron plays an important role in oxygen binding, electron transport, and as a cofactor for the function of many enzymes [15]. However, this highly transitional state of iron renders it toxic when present in its free form. Excess iron can catalyze the conversion of H_2_O_2_ to hydroxyl radical by undergoing Fenton reaction. Hydroxyl radicals, the most reactive oxygen species, cause lipid peroxidation and DNA strand breaks, thereby promoting the oxidative damage in tissues leading to cellular aging and death termed ferroptosis [16].

Iron homeostasis in the body is regulated primarily at the level of intestinal absorption of dietary heme or non-heme iron [17]. Although a minor amount of iron is lost every day from the body through sloughing of intestinal mucosal cells, urinary excretion and menstruation in women, there is no active mechanism for iron excretion [17]. Aging is associated with increase in non-heme iron such as ferritin or hemosiderin iron in different tissues [18,19,20]. As iron progressively accumulates with age, cellular iron retention is further exacerbated by dietary iron intake, genetic factors, repeated blood transfusions, and in certain pathological conditions. The mammalian target of rapamycin (mTOR), a serine/threonine kinase that plays a crucial role in aging, is inhibited by rapamycin through iron chelation mediated by hepcidin [21]. In addition, age-associated persistent accumulation of senescent cells in tissues exhibit proinflammatory cell fate and aberrant iron homeostasis [22]. In fact, the abundance of senescent cells has been found to influence the iron levels in aging tissue by impairing ferritinophagy, a lysosomal process that promotes ferritin degradation and ferroptosis [23]. Conversely, excess iron stimulates senescence in cells [24] causing organ dysfunction by producing reactive oxygen species leading to liver injury, diabetes, cardiac disorders, endocrine dysfunction, neurodegeneration, and ocular diseases.

## 3. Iron Overload in Aging Associated Diseases

**Figure 1:**
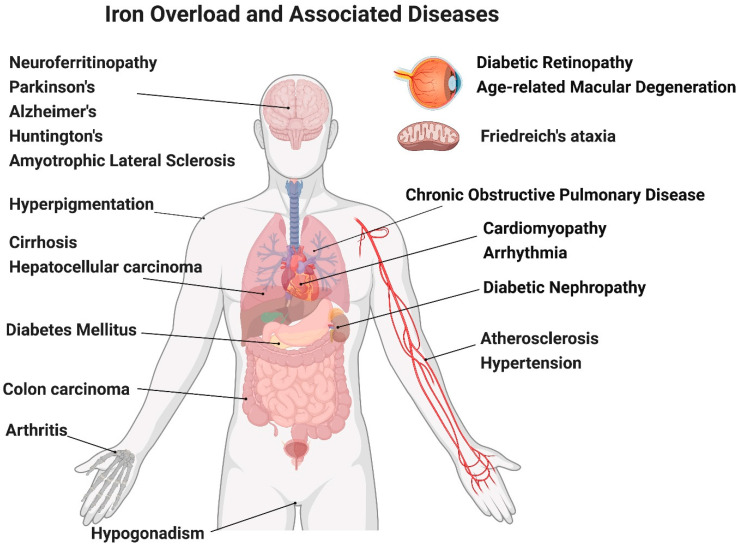
Illustration of iron overload associated diseases. Created with BioRender.com (accessed date: 15 April 2022).

The detrimental effects of iron in the aging process are attributed to abnormal cellular iron absorption, trafficking, or storage, affecting different systems of the body as shown in Figure 1. 

Mutation in any of the genes involved in iron homeostasis, namely HFE (High Fe), hemojuvelin, hepcidin, ferroportin, or transferrin receptor, leads to a genetic disorder called hereditary hemochromatosis (HH) [25]. Hemochromatosis patients and mouse models accumulate iron drastically with age in various organs as indicated in Table 1.

Iron overload in hemochromatosis manifests as arthritis [46], cirrhosis [47], hepatocellular carcinoma [48], diabetes [49], hypogonadism [50], or cardiomyopathy [51]. Similarly, mutation in the iron storage protein L-ferritin contributes to neuroferritinopathy (NF), which belongs to a heterogenous group of disorders called neurodegeneration with brain iron accumulation (NBIA) and presents an accelerated aging process with signs of early neurodegeneration and motor coordination deficits [52]. This disorder is characterized by accumulation of iron in the basal ganglia, cerebellum, and motor cortex of the brain with symptoms of chorea, dystonia, and cognitive impairments that worsen with age [53]. In aceruloplasminemia and Friedreich’s ataxia, mutation in ferroxidase ceruloplasmin or in mitochondrial iron storage protein frataxin respectively can lead to iron overload in a multitude of organs [54].

Apart from genetic factors, there is growing evidence that neuronal iron accumulation due to aging or dietary exposure has a substantial role in degenerative diseases including Parkinson’s [55], Alzheimer’s [56], Huntington’s [57], and amyotrophic lateral sclerosis [58]. The misfolding and aggregation of neuronal proteins like α-synuclein, amyloid beta (Aβ), and Tau is a common hallmark of many neurodegenerative disorders. Iron has been found to enhance aggregation of α-synuclein [59], Aβ [60], or Tau [61] either directly by iron binding to the amyloidogenic proteins or indirectly by iron-mediated aggregation through reactive oxygen species production. In addition, several clinical studies indicate that iron overload condition with high levels of serum ferritin at admission during stroke is associated with increased brain damage and worse outcome induced by ischemic stroke [62,63]. Iron is also involved in the pathogenesis of age-related macular degeneration (AMD) with strong experimental evidence indicating that retinas of AMD patients contain more iron than retinas of healthy subjects [64]; however, further studies are warranted to determine if iron is a cause or a consequence of AMD.

Patients with hypertension along with iron overload display sympathetic activation characterizing high blood pressure [65]. Similarly, increased serum ferritin is found to be a significant predictor for developing hypertension in middle-aged men [66]. We have reported that iron overload in mouse induces renin angiotensin system, a critical signaling pathway that regulates blood pressure [67]. Iron deposition in the heart often causes arrhythmias, progressive systolic dysfunction, cardiac hypertrophy, and cardiomyopathy [68]. Intraplaque hemorrhage in atherosclerosis may contribute to further free iron releasealtering the inflammatory and lipid metabolism, which in turn may accelerate atherogenesis leading to myocardial infarction. Thus, iron chelation plays as a potential therapeutic role in managing cardiovascular diseases [69,70].

Type 2 diabetes is a common complication in hemochromatosis patients with excess iron [71]. In fact, moderately elevated iron and ferritin levels, and a lower ratio of transferrin receptors to ferritin are associated with an increased risk of type 2 diabetes independent of other known diabetes risk factors [72]. Iron-catalyzed hydroxyl radical formation is considered to contribute to insulin resistance initially and reduced insulin secretion subsequently resulting in the development of type 2 diabetes [73]. Iron also accelerates end organ damage during diabetes by augmenting the progression of diabetic retinopathy and nephropathy in mouse models of diabetes with iron overload [67,74]. However, further research is needed to corroborate the causative role of iron in diabetic retinopathy and nephropathy progression in human patients.

Carcinogenicity of iron due to its prooxidant activity leading to oxidative damage has been shown in animal models [75] as well as in human patients [76]. Hence majority of hemochromatosis patients with iron overload present with cirrhosis and hepatocarcinoma. Iron is suggested as a risk factor for many types of cancer [77] including liver [78], colorectal [79], breast [80], and lung cancer [81]. Heme iron derived mainly from intake of red meat can induce cancer by acting as a nitrosating agent forming carcinogenic N-nitroso compounds [82]. In addition to solid tumors, recent evidence indicates iron overload in myelodysplastic syndrome (MDS) and in acute myeloid leukemia (AML), contributed by factors inherently associated with these diseases and due to multiple blood transfusions [83]. The status of iron overload has been shown to have a prognostic impact both in MDS and AML patients making iron and iron regulatory proteins an essential therapeutic target which can be explored further for chelation as well as for targeted delivery of anti-cancer drugs.

Iron present in cigarette smoke is an environmental factor with a strong causative link to pulmonary damage [84]. Iron-responsive element-binding protein 2 (IRP2) has been implicated in the development and progression of chronic obstructive pulmonary disease (COPD) [85], making IRP2 a powerful therapeutic target. The primary rheumatic manifestations of iron overload in hemochromatosis are arthropathy and osteoporosis [86]. Synovial iron accumulation is also found in patients with hemochromatosis and rheumatoid arthritis [87]. Iron deposition and defects in cartilage and immune function have been found to contribute to the development of arthritis in hemochromatosis patients.

A direct relationship between aging, and susceptibility and severity to infectious diseases is well known [88]. Iron is one such factor that accumulates with aging and also contributes to developing infections as elevated iron levels promote microbial growth, and impede inflammatory responses with consequent defects in bacterial clearance [89]. Peripheral blood monocytes during iron overload also secrete reduced amounts of tumor necrosis factor TNFα in response to lipopolysaccharide (LPS) [90]. Innate immunity effectively restricts iron availability to invading microbes by iron sequestration and improving the ability of phagocytes to kill microbes. Iron overload leads to dysfunctional bacterial phagocytosis due to destabilization of the secondary lysosomes in macrophages and decreased phagolysosomal fusion in peripheral blood monocytes [91]. The host’s iron status thus has a potential impact on the susceptibility and severity to infectious diseases, and conversely, infections also alter host iron homeostasis [92].

## 4. Ironing out the Aging

Since iron plays an important role in the aging process, reducing iron levels can be a significant therapeutic option to prevent the incidence and progression of chronic diseases. The most common treatment to reduce iron levels in hemochromatosis patients is repeated phlebotomy and patients continue having normal life expectancy as long as their iron levels are adequately managed before developing cirrhosis or diabetes [93]. Iron chelators in current clinical use are siderophores derived from micro-organisms, synthetic chelators and natural chelators. Desferrioxamine, also referred as Deferoxamine mesylate or Desferal^®^, is the most common siderophore to treat patients with iron overload. Although long term use of deferoxamine therapy has been demonstrated to be safe, it requires parenteral administration due to poor oral bioavailability [94].

Deferiprone or Ferriprox^®^ is a widely used synthetic iron chelator with comparable efficacy to deferoxamine. Deferiprone is used orally and can penetrate membranes easily allowing iron removal from tissues [95]. Deferasirox or Exjade^®^ is an oral chelator that has been approved for treating chronic iron overload due to blood transfusion [96]. Salicylaldehyde isonicotinoyl hydrazone (SIH) is a small molecule lipophilic iron chelating agent from the group of pyridoxal isonicotinoyl hydrazone (PIH) analogues that firmly binds ferric ions from the cellular labile iron pool and possesses a better ratio of chelation efficiency to toxicity among various iron chelators including deferoxamine, deferiprone, and deferasirox [97]. Thiosemicarbazones possess iron chelating activity and inhibit ribonucleotide reductase (RR), an iron-dependent enzyme that catalyzes the rate-limiting step in DNA synthesis [98]. Triapine^®^ (3-aminopyridine-2-carboxaldehyde thiosemicarbazone [3-AP]), Dp44mT (di-2-pyridylketone-4,4,-dimethyl-3-thiosemicarbazone) and DpC (di-2-pyridylketone 4-cyclohexyl-4-methyl-3-thiosemicarbazone) are some of the thiosemicarbazones that act as iron chelators and are widely used as anticancer drugs [99,100,101]. Dexrazone is an iron chelator belonging to the class of bis(2,6-dioxopiperazines) and clinically approved as a cardioprotective agent to treat doxorubicin-induced cardiotoxicity [102]. Clioquinol, an 8-hydroxyquinoline analogue, is a small lipophilic chelator of iron, copper and zinc showing substantial potential for the treatment of neurodegenerative diseases [103]. In addition, clioquinol is found to inhibit the aging-associated mitochondrial protein Clock-1, CLK-1 (human homologue Coenzyme Q7 or CoQ7) [104]. Long term aspirin use has been observed to lower serum ferritin in patients [105] and is reported to extend the lifespan in *C. elegans* [106]. Ibuprofen [107], doxycycline [108], metformin [109,110], clofibrate [111,112,113], fenofibrate [114,115], and enalapril [116,117] have all been found to have iron chelating activity and can increase longevity by protecting against oxidant-induced damage.

Natural chelators derived from spices and plants have also been investigated for their therapeutic properties. Curcumin from the dietary spice turmeric or *Curcuma longa* [118], and floranol from the roots of *Dioclea grandiflora* in Brazil [119] are found to efficiently chelate iron. Phytic acid in soy protein is an inhibitor of nonheme iron absorption and incorporating soy in the diet can reduce iron stores [120]. In addition, soy isoflavone genistein also can chelate iron and is indicated to have a therapeutic role in obesity and Type II diabetes [121]. Quercetin, a plant flavonoid found in red wine, green tea, apples and berries, binds Fe^2+^ more strongly than the well-known ferrous chelator ferrozine [122]. Epigallocatechin-3-gallate (EGCG) in green tea [123], Baicalein and its glycoside baicalin found in the Chinese herb *Scutellaria baicalensis* Georgi [124], Apocynin derived from the Ayurvedic Indian medicinal herb *Picrorhiza kurroa* [125], *Mucuna pruiens* [126], Kolaviron from African seeds *Garcinia kola* [127], and tannic acid found in gallnut, wine, and tea [128] have all been found to have iron-chelating properties. However, caution is required in using herbal remedies as iron chelators since the effect is not attributable to a specific chemical component and further research on the safe effective dosage and organ toxicity is warranted. Careful dosing of iron chelator is important to prevent the opposite concern of iron deficiency anemia which can lead to a wide range of other symptoms. Apart from siderophores, synthetic and natural iron chelators listed in Table 2, reduced intake of iron-rich food like red meat [129] and calorie restriction in general [130] have been found to reduce body iron levels and increase healthspan and lifespan [4,131].

## 5. Conclusions

There is astounding growing evidence between iron accumulation and aging. As iron naturally accumulates through life, various factors including excessive dietary intake, genetic factors, blood transfusions, and pathologic conditions exacerbate its progression. Interestingly, iron accumulation has been linked to sympathetic activation, signaling pathways that regulate blood pressure, autophagy, and senescence, thereby contributing to cardiovascular, neurodegenerative, metabolic, and cancer pathogenesis. In addition to the prooxidant signaling, the effects of iron independent of the oxidative stress pathway should also be considered. The degree of iron overload evaluated by measuring serum ferritin and transferrin saturation are inexpensive and helpful but are non-specific. Liver iron concentration determined from biopsy is invasive and has high sampling variability. Superconducting quantum interference device (SQUID) to estimate the magnetic susceptibility of the liver is generally accurate but the equipment is not commonly available yet. Magnetic resonance imaging (MRI) assessment of tissue iron has become the de facto method of evaluation in chronically iron overload patients. Clinical studies aimed at analyzing reliable biomarkers to detect pathological tissue iron overload could be a promising diagnostic tool in the future.

Due to devastating effects of iron accumulation, repeated phlebotomy, iron chelation, and diet restriction are mainstay therapeutic opportunities to prevent the iron toxicity. In addition to siderophores derived from micro-organisms and synthetic iron chelators, several natural iron chelators derived from herbs have been identified as therapeutic targets and continue to be heavily investigated. Iron chelators are also able to cross the blood brain barrier and remove excess iron from various regions of the brain [132]. Clinical trials exemplified the benefit of deferiprone in significantly attenuating neuronal loss in patients with Parkinson’s disease and Friedrich ataxia [133,134]. Type and severity of iron overload, dosage, treatment duration, and cost are critical factors that must be taken into consideration in selecting a chelator. Iron is chelated from different organs at different rates with hepatic iron overload usually improving more rapidly than other organs depending on the intensity of chelation. Therefore, the major challenges are to develop a safe and feasible drug that can reduce iron in organ-specific manner while maintaining systemic iron balance. In addition to chelation therapy, alternative approaches like targeting the regulation of proteins involved in iron metabolism and novel methods to induce ferroptosis demands further investigation. Preclinical studies in mouse models of iron overload have shown that long acting hepcidin analogs, also referred to as minihepcidins [135,136], and antisense oligonucleotides targeting TMPRSS6, a metalloprotease that inhibits endogenous hepcidin production [137], effectively decrease iron load, improve erythropoiesis, and increase hemoglobin concentrations, thus showing merit for further clinical investigation. Future studies on senescence, autophagy and ferroptosis to determine the temporal sequence of the molecular and cellular signaling events during chronic iron accumulation are necessary to develop effective therapies that prevent iron toxicity, and promote health and longevity.

## Figures and Tables

**Table 1:** Iron values in normal and hemochromatosis human and mouse samples.

Iron	Normal	Hemochromatosis
Human Serum	0–14 year old: 16–128 µg/dL [26]	HFE-hemochromatosis 212.65 ± 30.82 mg/dL [27]
	14–19 year old: 31–168 µg/dL (Male), 20–162 µg/dL (Female) [26]	Hemojuvelin-hemochromatosis 225–315 μg/dL [28]
	Adults: 50–150 µg/dL or 9–27 μmol/L [29]	Hepcidin/Ferroportin-hemochromatosis 284 μg/dL [30]
Human Liver	0.2–2 mg/g or 3.6–36 μmol/kg [31]	HFE-hemochromatosis 20 mg/g [31]
		Hemojuvelin-hemochromatosis 411–429 μmol/g [28]
		Hepcidin/Ferroportin-hemochromatosis 200–500 μmol/g [32]
Human Brain	Quantitative susceptibility mapping (parts per million, PPM) [33] Caudate: 0.108 ± 0.002 Putamen: 0.095 ± 0.002 Pallidum: 0.190 ± 0.003 Cortex: 0.041 ± 0.001	HFE-hemochromatosis Quantitative susceptibility mapping (parts per million, PPM) [33] Caudate: 0.108 ± 0.002 Putamen: 0.104 ± 0.003 * Pallidum: 0.187 ± 0.004 Cortex: 0.042 ± 0.001
	Putamen: [34] 50–59 years: 777 ± 267 μg/g 80–89 years: 1155 ± 363 μg/g	
	Globus pallidus: [34] 50–59 years: 577 ± 266 μg/g 80–89 years: 1062 ± 526 μg/g	
	Caudate nucleus: [34] 50–59 years: 533 ± 268 μg/g 80–89 years: 743 ± 360 μg/g	
Human Retina	0–35 years: 76.5 ± 9.15 µg/g [35] >65 years: 116 ± 9.73 µg/g [35]	
Mouse Serum	10 week old: 291 ± 9 μg/dl [36]	HFE-hemochromatosis 323 ± 24 μg/dL [36]
	14 week old: 26.20 ± 1.597 μmol/L [37]	Hemojuvelin-hemochromatosis 48.50 ± 1.682 μmol/L [37]
	24 week old: 34.9 ± 8.7 μmol/L [38]	Hepcidin/Ferroportin-hemochromatosis 39 ± 10 μmol/L [39]
Mouse Liver	10 week old: 255 ± 23 μg/dL [36]	HFE-hemochromatosis 2071 ± 450 μg/dL [36]
	≤6 months: 50.6 ± 1.66 μg/g [40]	Hemojuvelin-hemochromatosis 6070 ± 411.3 μg/g [37]
	≥16 months: 65.6 ± 2.35 μg/g [40]	Hepcidin/Ferroportin-hemochromatosis 1119 ± 176 μg/mg [38]
Mouse Lung	36 week old: ~0.6 μmolg [41]	HFE-hemochromatosis ~0.9 μmol/g [41]
	25–35 week old: 90–100 μg/g [42]	Hepcidin-hemochromatosis ~400 μg/g [42]
	36 week old: ~250 μg/g [43]	Ferroportin-hemochromatosis ~2300 μg/g [43]
Mouse Heart	1 month old: ~30 μg/g [44]	HFE-hemochromatosis ~75 μg/g [44]
	2.5 month old: ~5 μmol/g [45]	Hemojuvelin-hemochromatosis ~20 μmol/g [45]
	12 months old: ~50 μg/g [44]	Ferroportin-hemochromatosis ~9 ng/mg [38]

* indicates *p* < 0.05.

**Table 2:** List of Iron Chelators.

Siderophores	Synthetic Chelators	Natural Chelators
Deferoxamine mesylate	Deferiprone Deferasirox Salicylaldehyde isonicotinoyl hydrazone (SIH) Thiosemicarbazones (triapine, Dp44mT, DpC) Dexrazoxane Clioquinol Aspirin Ibuprofen Doxycycline Metformin Clofibrate Fenofibrate Enalapril	Curcumin Floranol Phytic acid Soy isoflavone genistein Quercetin Epigallocatechin-3-gallate (EGCG) Baicalein/Baicalin Apocynin Mucuna pruiens Kolaviron Tannic acid
